# A modeling study to inform screening and testing interventions for the control of SARS-CoV-2 on university campuses

**DOI:** 10.1038/s41598-021-85252-z

**Published:** 2021-03-15

**Authors:** Ben Lopman, Carol Y. Liu, Adrien Le Guillou, Andreas Handel, Timothy L. Lash, Alexander P. Isakov, Samuel M. Jenness

**Affiliations:** 1 Department of Epidemiology, Rollins School of Public Health, Emory University, Atlanta, GA 30322 USA; 2 Department of Research and Public Health, Reims Teaching Hospitals, Robert Debré Hospital, Reims, France; 3 College of Public Health, University of Georgia, Athens, GA 30602 USA; 4 School of Medicine, Emory University, Atlanta, GA 30322 USA

**Keywords:** Viral infection, Epidemiology

## Abstract

University administrators face decisions about how to safely return and maintain students, staff and faculty on campus throughout the 2020–21 school year. We developed a susceptible-exposed-infectious-recovered (SEIR) deterministic compartmental transmission model of SARS-CoV-2 among university students, staff, and faculty. Our goals were to inform planning at our own university, Emory University, a medium-sized university with around 15,000 students and 15,000 faculty and staff, and to provide a flexible modeling framework to inform the planning efforts at similar academic institutions. Control strategies of isolation and quarantine are initiated by screening (regardless of symptoms) or testing (of symptomatic individuals). We explored a range of screening and testing frequencies and performed a probabilistic sensitivity analysis. We found that among students, monthly and weekly screening can reduce cumulative incidence by 59% and 87%, respectively, while testing with a 2-, 4- and 7-day delay between onset of infectiousness and testing results in an 84%, 74% and 55% reduction in cumulative incidence. Smaller reductions were observed among staff and faculty. Community-introduction of SARS-CoV-2 onto campus may be controlled with testing, isolation, contract tracing and quarantine. Screening would need to be performed at least weekly to have substantial reductions beyond disease surveillance. This model can also inform resource requirements of diagnostic capacity and isolation/quarantine facilities associated with different strategies.

## Introduction

In an unprecedented response to the COVID-19 pandemic, schools (including institutions of higher education) in almost every nation closed in the first half of 2020^1^. For boarding institutions like universities, this involved both transitioning classes into online teaching as well as closing dormitories by sending students off-campus. School closure as a non-pharmaceutical intervention has been aimed at reducing contact among students, family members, teachers, and school staff^2^. Closure is thought to be an effective means of reducing disease transmission based on the understanding that younger people are important in transmission of respiratory viruses, like influenza. Closure of schools early in a pandemic may often be more impactful than delayed closing^2^. According to UNESCO, approximately 70% of the global student population has been affected with closures of pre-school, primary, secondary, and higher education institutions^1^. Since SARS-CoV-2 infections are particularity severe among older adults while younger people still get infected and transmit^3^, university populations are unique in their degree of mixing across these age groups. Prior to the emergence of SARS-CoV-2, contact data on transmission of influenza, and other respiratory virus, provided the basis of current recommendations.

Universities are important and unique in that they are frequently residential, involve students traveling long distances to attend, and are assets to their regional economies. University administrators are now facing decisions regarding if and how to safely return students, staff, and faculty to campus. As of the end of July 2020, approximately one-third of US universities are planning for primarily in-person instruction for Fall 2020^4^. Universities considering campus re-opening need to estimate the resources necessary to interrupt and mitigate on-campus transmission by projecting the number of possible cases, needs for screening and testing, and boarding requirements for persons needing isolation and quarantine.

To provide a framework to evaluate these questions, we developed a susceptible-exposed-infectious-recovered (SEIR) type of deterministic compartmental model. This model captures the transmission process and can therefore estimate the direct and indirect (i.e. transmission-mediated) effects of control strategies. For example, through model simulations, we estimated how testing and identifying SARS-CoV-2-infected students results in them being isolated, their contacts being quarantined, as well as all the infections averted by preventing the chains of transmission that would have otherwise occurred. While our goals were to support pandemic planning at our university, we offer a flexible modeling framework through an RShiny application to inform the efforts of similar university campuses interested in exploring the relative impacts of screening, testing and tracing on COVID-19 cases. Since the initial launch of our application in June, 2020, a number of campus models have been constructed^5–9^. In addition to our focus on screening and testing-based interventions, our model is unique in that we offer an accompanying online platform that allows decision-makers to input parameters appropriate for their respective campuses to guide their intervention strategy and preparedness.

## Methods

**Figure 1:**
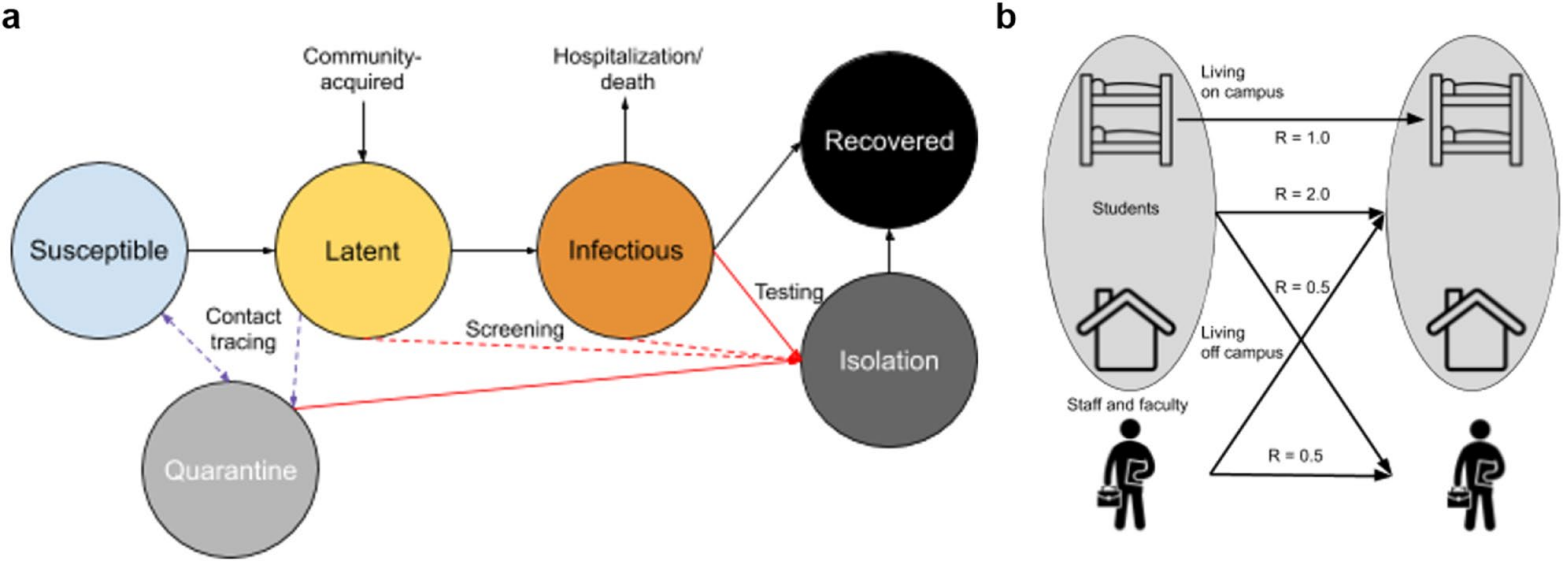
Schematic of (**a**) disease structure and (**b**) student and staff/faculty transmission pathways.

We developed a dynamic model of transmission of SARS-CoV-2 among students, staff and faculty, Fig. 1a represents the schematic of disease structure. We parameterized the model for our institution, Emory University, a medium-size private university in Atlanta, Georgia with 15,000 students and 15,266 staff and faculty. The model can be applicable to other colleges and universities; a public web application allows for key initial conditions and model parameters, such as student and staff population sizes and level of community risk, to be varied as background conditions change (https://epimodel.shinyapps.io/covid-university/). The main features and assumptions are described in the following sections. Table 1 provides a full list of all parameter values; the model equations are shown in SI.I.

**Table 1:** Model parameters and ranges.

Parameter	Value	Range	Distribution	Symbol	Source
**Populations**					
Total students	15,000				Univ. admin
Students living on campus	4,500				Univ. admin
Staff and faculty	15,266				Univ. admin
**Natural history and clinical**					
Latent period (days)	3	2 to 4	Gamma	$\alpha$	^17^
Infectious period (days)	7	6 to 8	Gamma	$\gamma$	^29^
Proportion severe—students	0.0224	0.0133 to 0.0456	Beta	${p}_{sev,stu}$	^15^
Proportion severe—staff/faculty	0.055	0.0327 to 0.1122	Beta	${p}_{sev,saf}$	^15^
Proportion fatal—students	0.0006	0.0003 to 0.0014	Uniform	${p}_{death,stu}$	^15^
Proportion fatal—staff/faculty	0.0052	0.0029 to 0.0105	Uniform	${p}_{death,saf}$	^15^
Proportion symptomatic—students	0.35	0.27 to 0.43	Beta	$1-{p}_{asy,stu}$	^3^
Proportion symptomatic—staff/faculty	0.51	0.41 to 0.59	Beta	$1-{p}_{asy,saf}$	^3^
**Transmission**					
R_0_: students to students	2	0.7 to 2.5	Uniform	${R0}_{stu,stu}$	^30^
R_0_: on campus students to other on campus students	1	0.3 to 1.4	Uniform	${R0}_{on,on}$	^12,26^
R_0_: Staff/faculty to student; staff/faculty to staff/faculty	0.5	0.15 to 0.7	Uniform	${R0}_{saf}$	
Daily per capita community case incidence	0.00005	0.000025 to 0.0001	Beta	daily_new_case	^18^
Under-reporting factor for community infections	10			under_report	^19^
Efficacy of face-coverings and social distancing	0.35	0.18 to 0.43	Beta	eff_npi	^14^
**Testing and quarantine**					
Time from onset of infectiousness to testing (days)	2	7 to 1	Uniform	test	Intervention scenario
Screening interval (days)	30	120 to 1	Uniform	scr	Intervention scenario
Duration of quarantine (days)	14			δ	Univ. admin
Number of contacts per case	2	1 to 7	Uniform	contacts	Univ.admin
Proportion of contacts reached	0.75	0.5 to 1	Uniform	p_cont	
Proportion experiencing ILI symptoms per day	0.00333	0.003 to 0.003667		ili	^20,21^
PCR sensitivity—day 2 of infectiousness	0.75	0.6 to 0.83	Beta	${se}_{2}$	^22^
PCR sensitivity—day 4 of infectiousness	0.8	0.7 to 0.85	Beta	${se}_{4}$	^22^
PCR sensitivity—day 7 of infectiousness	0.75	0.65 to 0.8	Beta	${se}_{7}$	^22^

### Population structure and transmission

We modeled three distinct population groups with different degrees of interactions among them: students living on-campus; students living off-campus; and staff and faculty (Fig. 1b). R0 values for SARS-CoV-2 were estimated to be between 2.2 and 6.5 in an early systematic review and further declared as between 2 and 4 by the World Health Organization (WHO)^10^. Our group estimated R0 to be 3.5 in the state of Georgia before lockdown measures were in place^11^. Without definitive estimates for R0 for on-campus students, off-campus students and staff in an unmitigated scenario, we use estimates from the general community (R0 = 3.5) to parameterize transmission from all students and staff to on-campus students. We further assume that the risk of transmission from the campus community to off-campus students is lower than the risk of transmission from campus community to on-campus students. This assumption is based on evidence of relative risk of infection comparing on-campus and off-campus students from a campus-outbreak of H1N1 in 2009 where off-campus students were at a substantially lower risk^12^ and that on-campus students live in congregate settings that are considered higher risk for transmission. Based on this logic, we parameterize transmission from all students and staff to off-campus students as R0 = 2.5 and parameterize transmission from staff to staff and all students to staff as R0 = 0.5, respectively.

Universities are planning an array of measures to limit transmission on campus. These may include mask-wearing; other personal protective equipment; smaller class sizes; staggered class times; enhanced cleaning protocols; enhanced hygiene; canceling large social gatherings; fewer students living in dorms and restricting off-campus movements^13^. We lack data on the efficacy of all these non-pharmaceutical interventions, especially in this specific population, but we assumed that they will have an effect on transmission. We parameterized these non-pharmaceutical controls based on a systematic review of the effect of social distancing and face coverings^14^ (and assuming 50% compliance), and we explored a range of values around this parameter.

Staff and faculty had a higher risk of severe illness and death (given infection) than students, based on accumulating evidence of age-differences in the case-fatality rate^15^. We then standardized using the student and staff/faculty age-structure at our institution. [For a full list of parameter values, see Table 1]. We further assumed that a fraction of cases were asymptomatic and that the probability of symptoms was greater for staff/faculty given their older age distribution than students. We assumed that asymptomatic infected persons were as infectious as those with symptoms; this assumption may overestimate the real transmission rate in this group^16^. We assumed that infectiousness begins on the third day after infection; this latent period is shorter than the incubation period^17^ to represent pre-symptomatic transmission. We do not track transmission in the wider community explicitly, but incorporated introduction of virus onto campus from the community. We modeled this as a constant daily rate of infection being introduced on campus. In our model parameterization, this was based on confirmed COVID-19 cases in Fulton and Dekalb Counties that surround our institution in early June (around 100 per day) and the combined population of the two counties^18^. We further assumed that infection incidence was ten-times that of reported cases^19^. The model runs for a semester from the day classes start (August 26) to the end of term (December 19), or 116 days. We did not assume reduced transmission over traditional Fall or Thanksgiving breaks or consider alternative semester schedules.

### Intervention design

In the model, control was initiated by SARS-CoV-2 diagnostics. Infected persons can be identified by reverse transcription polymerase chain reaction (RT-PCR) through either screening or testing, defined as follows. *Screening* is a strategy in which students, staff, and faculty are tested at a given frequency ranging from weekly to once per semester regardless of the presence of symptoms. *Testing* is a strategy whereby symptomatic students, staff, and faculty present for clinical care and are tested using RT-PCR. We assumed a background level of persons with influenza-like symptoms caused by infections other than SARS-CoV-2^20,21^ who will test negative but are included in estimations for total number of tests needed. Those with COVID-19 who test positive are immediately isolated. However, we assumed that the diagnostic has imperfect sensitivity that varies based on what date of illness the test is performed^22^. There is evidence that PCR sensitivity varies over the course of infection, reaching a peak around day 7 of infection (or day 4 of infectiousness), then declines again. Therefore, we examined the impact of variation in the testing interval, defined as the average lag time between symptom onset and quarantine. Because infectiousness begins one day before symptom onset in the model, we simulated testing intervals ranging from a two-day to a one-week test delay. These testing scenarios are in the absence of any screening to isolate the causal effects of this more intensive intervention.

Following both screening and testing, those diagnosed positive for COVID-19 were immediately isolated in the model. Case isolation involves a complete reduction in their contact rate for the duration of infection. Positive test results also lead to contact tracing. Contact tracing was simulated for screening and testing strategies by assuming public health authorities could elicit 2 contacts per case detected with 75% of those successfully traced and quarantined. The number of contacts per case is based on data from Emory University’s ongoing contract-tracing program. Quarantine, like isolation, was modeled as a complete reduction in the contact rate for the duration of infection. Some of those quarantined contacts might have been incubating but are now no longer able to infect since they are under quarantine.

### Parameterization and analysis

In our base models, we simulated SARS-CoV-2 transmission and interventions for the Fall 2020 semester. Our main base model explored a range of NPIs but no screening- or testing-based control. Counterfactual scenarios then varied the screening and testing rates, and the completeness of contact tracing. Our primary outcomes were both active cases per day and cumulative cases across the semester. The model tracked both total cases in each campus group (students versus staff/faculty) as well as severe cases and COVID-related mortality. Given uncertainty in model parameters, we performed a probabilistic sensitivity analysis to estimate the range of credible outcomes for all scenario analysis. In the probabilistic sensitivity analysis, we took 1000 parameter draws using Latin Hypercube Sampling from the distributions in Table 1. Plots for scenario analysis describe outcome distribution for the 50th percentile accompanied by a range of 25–75th percentile of those samples.

We further present results comparing the base scenario and three probable intervention scenarios initially considered by Emory University: (1) base scenario with no testing or screening; (2) testing for symptomatic cases with a 4-day testing interval between onset of infectiousness and testing; (3) screening for all staff and students with 30-day screening interval and (4) a combination intervention with 4-day testing interval and 30-day screening interval. For all models, we assume implementation of NPIs reduces transmission by 35%^14^. Outcome distributions for the 2.5th and 97.5th percentiles from probabilistic sensitivity analysis are provided in Table 2. Finally, we then used partial rank correlation coefficient to determine how much the modeled variation in cumulative incidence among students and faculty/staff depends on random parameter values. We recommend that these results be interpreted qualitatively, since there is considerable uncertainty in these projections stemming from lack of precision of parameter inputs (e.g. true R0 in this population).

**Table 2:** Cumulative outcomes at end of the semester on medium size university campus (approx. 15,000 students and 15,000 staff/faculty).

	Base scenario	4-day test interval	30-day screen interval	Combined test and screen
**Students**				
Cumulative cases (n)	3068 (951–4854)	795 (252–1915)	1259 (348–3021)	532 (200–1188)
Peak daily cases (n)	413 (77–956)	25 (7–111)	59 (12–269)	11 (4–32)
Hospitalizations (n)	179 (39–427)	46 (10–166)	74 (15–248)	32 (8–95)
Deaths (n)	5 (1–14)	1 (0–5)	2 (0–7)	1 (0–3)
Isolated (n)	0 (0–0)	449 (134–1226)	561 (143–1354)	371 (133–888)
Isolated (max)	0 (0–0)	66 (18–257)	92 (19–327)	49 (17–135)
Isolated (days)	0 (0–0)	5605 (1643–16,059)	7001 (1739–18,058)	4582 (1636–11,221)
Quarantined (n)	0 (0–0)	1249 (231–4060)	1507 (259–4848)	1026 (212–3052)
Quarantined (max)	0 (0–0)	182 (31–821)	251 (37–1086)	137 (27–439)
Quarantined (days)	0 (0–0)	15,528 (2828–52,481)	18,993 (3124–64,665)	12,718 (2615–38,184)
**Staff and Faculty**				
Cumulative cases (n)	1063 (427–2094)	502 (208–1011)	596 (234–1238)	460 (185–909)
Peak daily cases (n)	102 (30–285)	17 (6–38)	31 (11–75)	12 (5–25)
Hospitalizations (n)	107 (30–296)	50 (13–150)	59 (16–174)	46 (12–132)
Deaths (n)	10 (3–29)	5 (1–4)	6 (2–16)	4 (1–12)
**Testing**				
Total performed (n)	0 (0–0)	14,213 (11,993–18,066)	116,020 (116,020–116,020	129,145 (127,568–131,164)
Per capita	0 (0–0)	0 (0–1)	4 (4–4)	4 (4–4)

Values are medians and 2.5 and 97.5th percentiles of 1000 model samples.

The model was built and simulated in the *EpiModel* package in the R statistical computing platform^23^; the *LHS* package was used to perform Latin Hypercube Sampling^24^. We also built an interactive web app for model exploration using the R Shiny framework. It can be accessed at https://epimodel.shinyapps.io/covid-university/. All code and parameters are publicly-available and can be accessed at https://github.com/lopmanlab/covid_campus_model .

No human subjects were involved in this work. We report a modeling study using only aggregate data from published sources.

## Results

**Figure 2:**
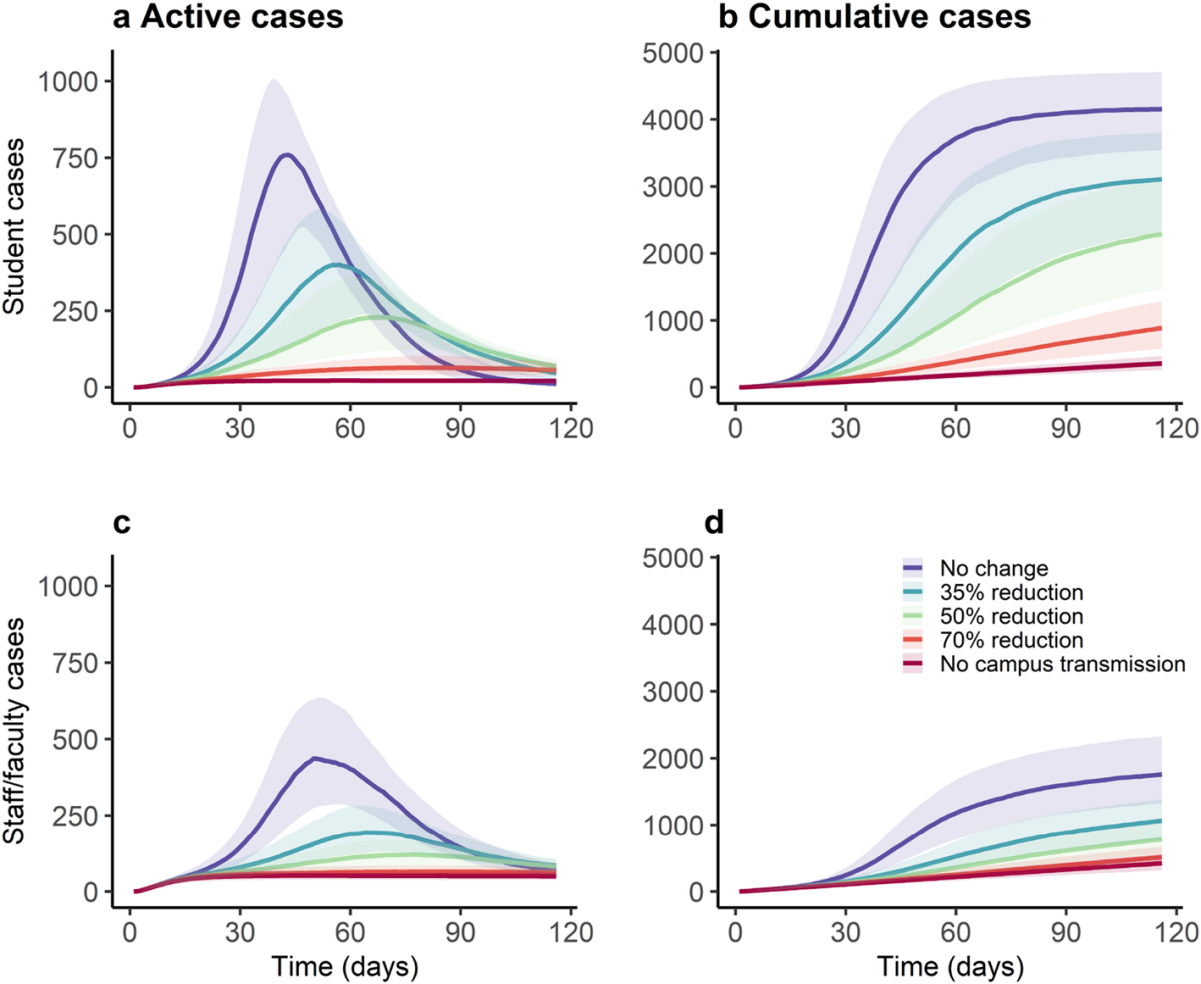
Effect of non-pharmaceutical interventions on COVID-19 active and cumulative cases among students (**a**,**b**) and faculty (**c**,**d**). Here we assume no baseline effects of non-pharmaceutical interventions, such that NPI effectiveness ranges from zero (no change) to complete (no on-campus transmission; only introduction from the community). Solid line represents the median of 1000 simulations using probabilistic sensitivity analysis, and shaded area represents the 25th-75th centile range.

Assuming an initial student population of 15,000 and staff and faculty of 15,266, we start by simulating transmission on campus (Fig. 2) in which no diagnostic control measures (testing, isolation, contact tracing, or quarantine) or NPIs (facemasks and social distancing) are in place. With an R0 of 3.5 for on-campus students and an R0 of 2.5 for off-campus students, case prevalence peaks at 760 cases (Range, 2.5th to 97.5th centiles: 263 to 1417) per day among students and 437 cases per day among staff/faculty (102 to 1004), resulting in a cumulative of 4154 (2468 to 5721) student cases and 1759 (794 to 3188) staff/faculty cases at the end of the semester. With our baseline levels of facemask and social distancing efficacy (35% reduction in transmission) but with no diagnostics, we estimate case prevalence peaks at 413 (77–956) per day among students and 102 (31–285) cases per day among staff/faculty, resulting in a cumulative of 3068 (951–4854) student cases and 1063 (427–2094) staff cases at the end of the semester. Furthermore, peak prevalence in both students and staff/faculty is delayed with increasing efficacy of NPIs. In students, peak prevalence is delayed from 45 days with no NPIs to 61 days with NPIs that reduce transmission by 35%. This number of symptomatic cases is substantially lower than the number of infections since we assume that 35% (27% to 43%) of infected students and 51% (41% to 59%) of infected staff are symptomatic, given infection^3^. We use this scenario as the baseline counterfactual for all subsequent comparisons.

**Figure 3:**
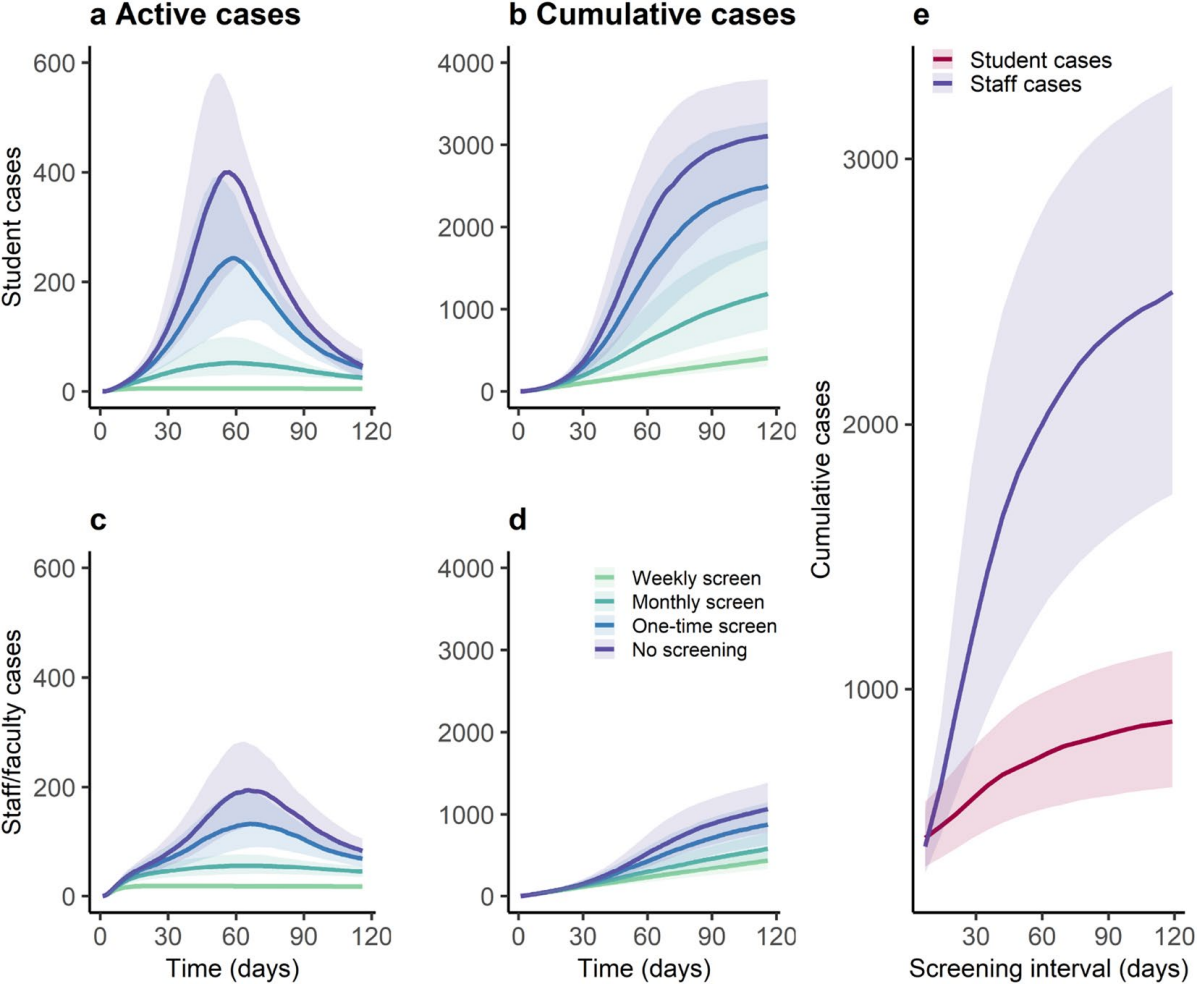
Impact of screening frequency on projected COVID-19 daily active and cumulative incidence among students (**a**,**b**) and staff/faculty (**c**,**d**) and cumulative student and staff/faculty cases over a sweep of varying screening intervals (**e**). Solid line represents the median of 1000 simulations using probabilistic sensitivity analysis, and shaded area represents the 25th-75th centile range. Legend highlights interventions of weekly screening, monthly screening and one-time screening compared with no screening.

We next use an NPI efficacy of 35% reduction as the base scenario and explored a wide range of screening intervals, from weekly to once during the semester (Fig. 3). One-time screening, whereby the population is tested on average once during the 4-month semester, reduced cumulative student incidence overall by 19%; monthly and weekly screening reduced cumulative student incidence by 59% and 87% respectively. For staff and faculty, one-time screening reduced cumulative incidence by 18%; monthly and weekly screening reduce cumulative incidence by 44% and 59% respectively. For students, the cumulative incidence ranged from 407 (150–847) with weekly screening to 2481 (667–4343) with one-time screening. For staff/faculty, the cumulative incidence ranged from 439 (173–873) with weekly screening to 873 (359–1731) with one-time screening. The screening interval did not impact the timing of peak prevalence for student or staff/faculty cases.

**Figure 4:**
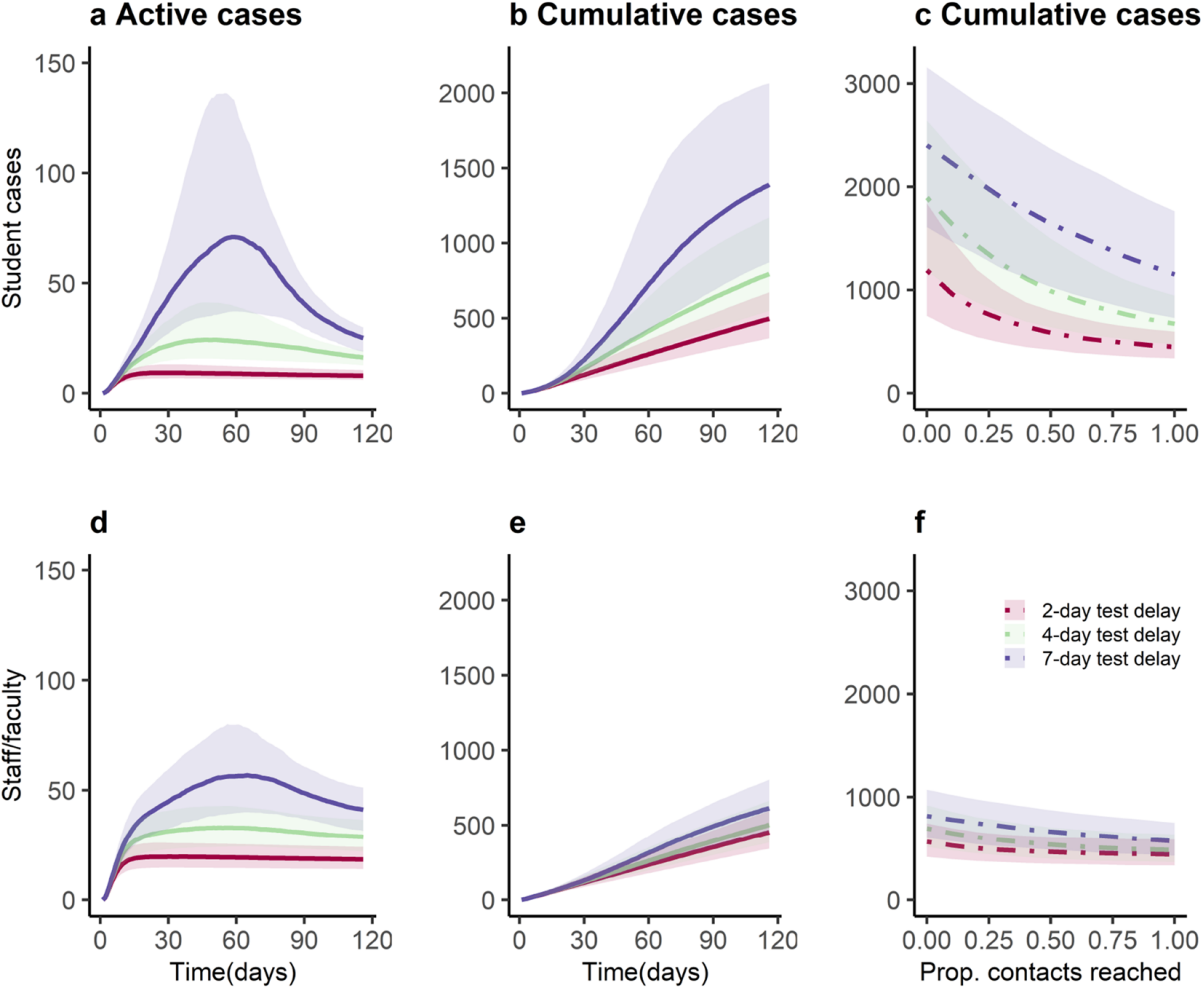
Impact of testing, contact tracing and quarantine at a range of testing delay intervals. Daily and cumulative COVID-19 incidence on university campus (**a**,**b**,**d**,**e**) and cumulative student and staff/faculty cases from 2-, 4-, 7- day test delay over a sweep of proportions of contacts reached (**c**,**f**). Based on ongoing contact-tracing program at Emory, we assume an average of two contacts elicited per case. Solid line represents the median of 1000 simulations using probabilistic sensitivity analysis, and shaded area represents the 25th-75th centile range.

We then consider a testing-only strategy where a positive test triggers contact tracing and quarantine. Here, with week-delayed testing (the least optimistic scenario), the expected cumulative incidence would be 1389 (355–3059) for students and 613 (245–1243) for staff/faculty. With a four-day delay testing interval, the expected cumulative incidence would be 795 (252–1915) for students and 502 (208–1011) for staff/faculty (Fig. 4). With a two-day delay testing interval, the expected cumulative incidence would be 496 (184–1053) for students and 452 (184–896) for staff/faculty. These scenarios represent a 55%, 74% and 84% reduction in cumulative incidence over the semester among students and a 42%, 53% and 57% reduction in cumulative incidence among staff & faculty. Increasing delays in testing interval from 2 to 7 days delays the timing of peak prevalence for student cases from 29 to 59 days and delays the timing of peak prevalence for staff cases from 36 to 65 days. The right panels in Fig. 4 also show the general relationship between “contact tracing” success and cumulative incidence assuming either a 2-day, 4-day, or 7-day delay in testing/quarantine following symptoms. Although the testing interval can reduce the cumulative incidence, the greater impact of this testing scenario is achieved by the number of contacts reached.

**Figure 5:**
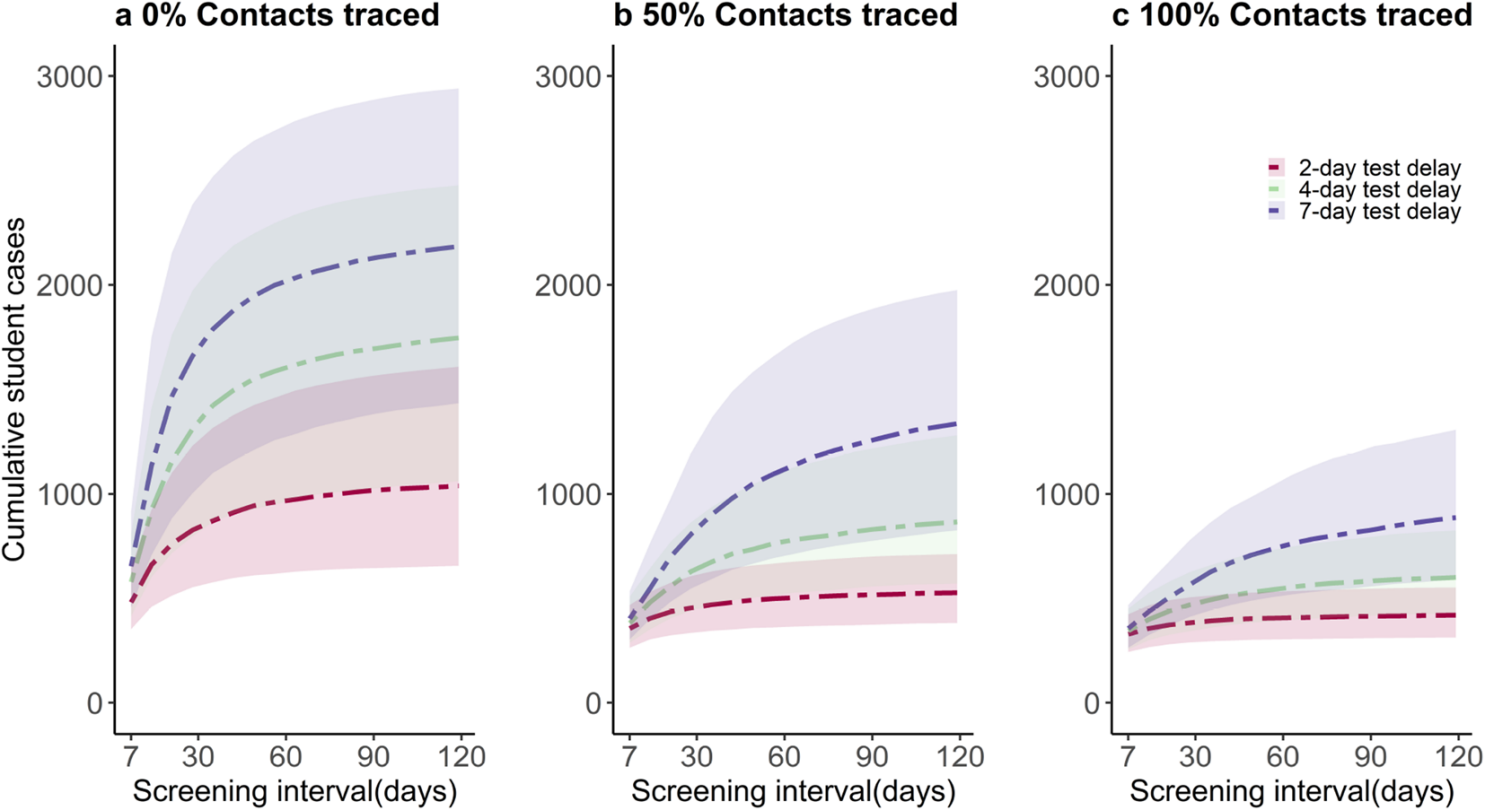
Impact of combined screening, testing and tracing of COVID-19 cases on cumulative cases among students. Panels represent scenarios with (**a**) 0%, (**b**) 50% and (**c**) 100% of contacts traced and successfully quarantined. Solid line represents the median of 1000 simulations using probabilistic sensitivity analysis, and shaded area represents the 25th-75th centile range.

In the final scenarios, we combined the testing and screening rates under different assumptions of contact tracing related to testing (Fig. 5). Our model scenarios below varied the interval for screening between 1 week (7 days) and 1 semester (116 days) and testing at 2-, 4- and 7-day delay, with the efficacy of contact tracing ranging from 0 to 100%. These figure panels show cumulative incidence at the end of the semester for students only. When combined with testing, screening generally had little effect unless it is performed at least monthly.

Under intervention scenarios, reductions in campus transmission corresponded with an increase in proportion of cases due to community transmission. In the base scenario, community transmission accounted for 6% and 39% of cases among students and staff. Comparatively, under 30-day screening interval, 4-day testing interval and combined 30-day screening interval, community transmission accounted for 20%, 35%, 52% cases among students, respectively, and 70%, 82%, 88% of cases among staff, respectively (SI. III).

### Partial rank correlation coefficient (PRCC)

**Figure 6:**
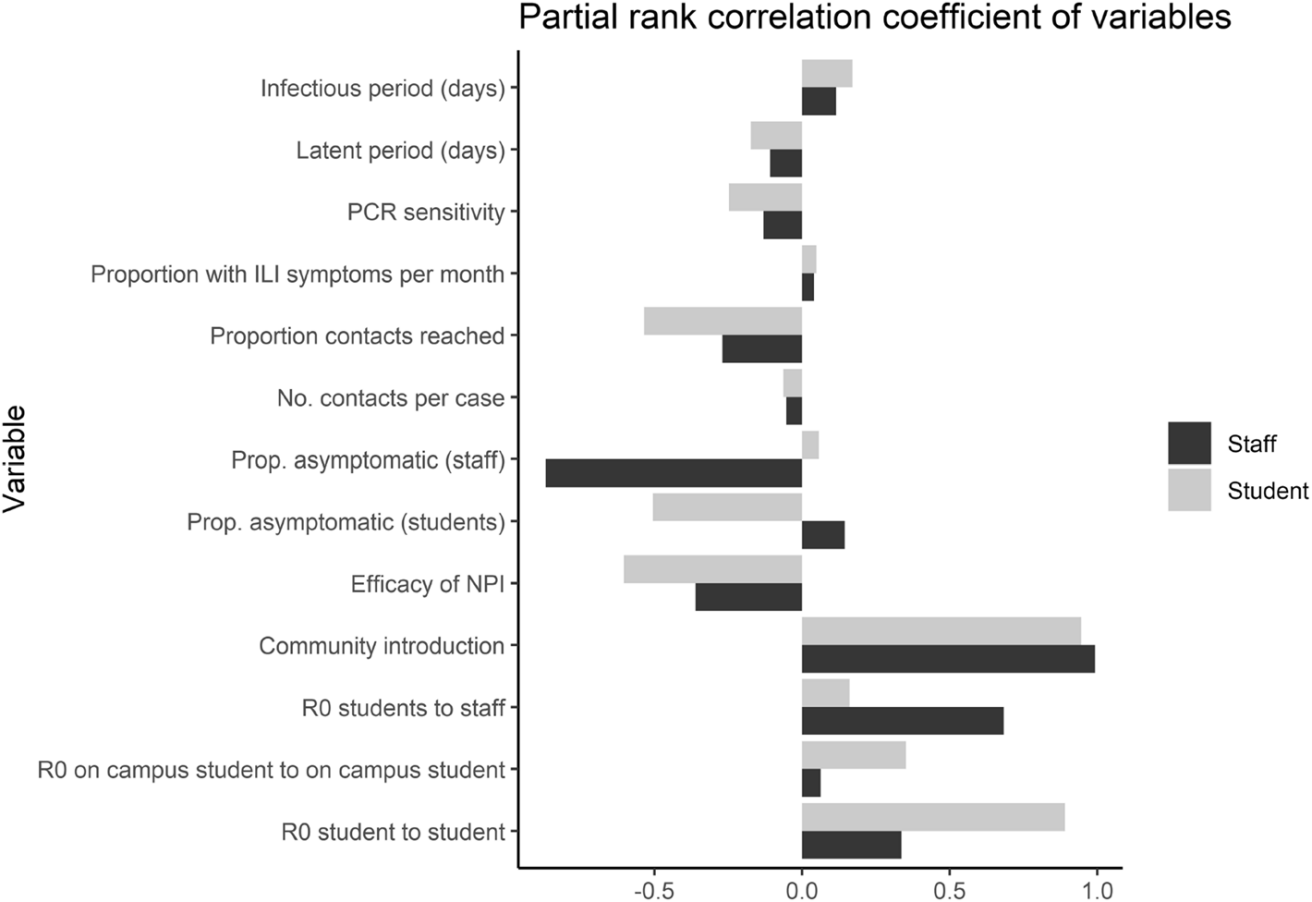
Partial rank correlation coefficient of key model inputs using outputs of cumulative student and staff/faculty cases in our model with combined 30-day screening and 4-day testing interval.

We used partial rank correlation coefficients (PRCC) to estimate the strength of association between the most relevant input parameters and the outputs of cumulative student and staff/faculty cases in our model with combined 30-day screening and 4-day testing interval (Fig. 6). We check assessed correlations between input parameters and outputs and found monotonic relationships (SI.IV), suggesting that PRCC is an acceptable method to summarize the pattern. We find that student cases are sensitive (PRCC ≤  − 0.5 or ≥ 0.5) to the following input parameters: proportion contacts reached (coef =  −0.52), proportion of asymptomatic student infections (coef = −0.51), efficacy of NPIs (coef =  −0.60), community transmission (coef = 0.95) and $R0$ for students (${R0}_{stu,stu}$) (coef = 0.89). Staff/faculty cases are sensitive to proportion of asymptomatic staff infections (coef = 0.87), community transmission (coef = 0.99) and $R0$ for staff (${R0}_{stu,saf}$) (coef = 0.68), but not sensitive to proportion of contacts reached, efficacy of NPIs, $R0$ for students or proportion of asymptomatic student infections. Neither student or staff cases were particularly sensitive to infectious period, latent period, number of contacts elicited per case or PCR test sensitivity.

## Discussion

We find that unmitigated transmission of SARS-CoV-2 in a university population of around 30,000 staff, faculty, and students would lead to thousands of illnesses, many hospitalizations, and likely some deaths. This is clearly an unacceptable outcome by administrators and the university community. Combined with measures to reduce transmission, a testing strategy whereby symptomatic students, staff, and faculty are identified, administered viral testing, and isolated may be effective at controlling transmission. The success of this strategy relies on contact tracing and quarantining most contacts of infected individuals. Screening would have to be performed at least monthly to have much of an impact on the course of the outbreak on campus and increases the sample collection and assay requirements considerably. However, because we assume the campus community is not a closed population and that there is an ongoing risk of importation of virus, there are considerable numbers of cases, mostly from community introductions, even under the most optimistic scenario. Furthermore, this scenario requires substantial financial and clinical capacity resources. Overall, we recommend that these results be interpreted qualitatively due to considerable uncertainty and lack of precision of parameter inputs.

There are a number of limitations to this modeling analysis. First, we used a deterministic, compartmental model that represents average population dynamics and behavior. An individual-based model would more comprehensively capture the effects of contact tracing, isolation and quarantine and would also allow the incorporation of other interventions such as reductions in class sizes and attendance limits for in-person events, but would require much more detailed data on these processes that we did not have. Second, we made simplifying assumptions for a number of model parameters. We acknowledge that all our model's transitions implicitly follow an exponential rate of flow. This is a simplifying assumption^25^, especially for isolation and quarantine, which are of fixed duration. We formulated the model in this manner for simplicity and consistency across transitions from different states, but recognize this as a limitation, though we expect that implementation of other distributions (e.g. gamma) would have modest effect on the findings. Furthermore, the efficacy of interventions such as smaller class sizes, staggered class times, use of face coverings, use of other protective equipment, and general behavior change are not separately considered^14^ because we lack empirical data about the efficacy of individual prevention and control measures aside from testing. If such data become available in campus populations or populations that can serve as a good proxy, model parameters can be refined. Relatedly, we used estimates of R0 for the general community, since we did not have data for university populations. Moving more students to off-campus housing had little effect on our projections because we assumed that transmission on-campus (R0 = 3.5) is only moderately higher than off campus (R0 = 2.5). This assumption is based on risk factor data on influenza-like illness among students during the 2009 H1N1 outbreak^12^ and is comparable to accruing evidence that residential and non-residential students have comparable risk of SARS-CoV-2 infection ^26^ Moreover, we assumed asymptomatically-infected individuals are equally infectious as symptomatically-infected individuals and factoring relative infectiousness of asymptomatic individuals is likely to reduce rates of infection. We also assumed a constant rate of introduction that is proportional to infection prevalence in surrounding communities, meaning the campus outbreak could not go extinct. Relatedly, we did not consider how campus-acquired infections lead to infections in the non-university community. And, while we rely on community reported case rates to inform the risk of importation onto campus, other indicators (e.g. deaths) can be used if the relative infection rate to that indicator is known (e.g. the infection fatality ratio).

Depending on levels of student, staff, and faculty behavior off-campus and the general prevalence in the surrounding community (the Atlanta metropolitan area in our model), this could be an under- or overestimate of risk on campus. We have not explicitly included a scenario in which all or a subset of students (e.g., those residing on campus) are screened upon return to campus. We did not include this because we reasoned that the continued risk of importation onto campus would overwhelm the risk from initial number infected. Given our assumptions that student prevalence is the same as among the general population, screening on return would have limited effect, but would increase requirements by ~ 4,500 to 15,000 tests, depending on the breadth of testing of the student body. Finally, we have not included seasonal effects whereby virus becomes more transmissible in Fall. Also, Emory, like many universities cancelled Fall and Thanksgiving breaks and shortened the semester, we therefore did not simulate the effects of travelling during break. We found the results to be quite sensitive to R0 as well as the proportion of contacts reached. R0 is an uncertain assumption of the model that could be refined with data from campus populations. The sensitivity of the results to the proportion of contacts reached highlight how important contact tracing is and should be a focus of mitigation efforts.

While we present numerical results for our university at a specific point in time, the model can be re-parameterized for other institutions and can be updated as the epidemiological situation shifts. We updated the model a number of times in discussions with our university leadership. Results from this framework have been influential in their ongoing decision-making. Community risk is a parameter that we updated as the local area incidence has increased. This is sure to change over time, and this will vary from place to place as the model is used by other university planners. While changing population sizes and testing rates is easily implemented, there are some caveats and limitations to consider when applying to other institutions. First, we assumed the capacity for contact tracing and high adherence to isolation and quarantine. That may be an overly optimistic assumption for some institutions, where capacity is limited. Second, the campus population structure may be quite different for non-residential or community colleges, for example. Finally, adherence to non-pharmaceutical interventions may be considerably different from institutions and over time. This is easily implemented in the model, but institutions require data on adherence to inform this.

Local data on reported incidence and estimates of under-reporting should be used to parameterize this value. Similarly, population immunity may increase as the pandemic progresses. Sero-survey data can inform the proportion of the university community that is immune upon campus opening. These parameters may be updated in our accompanying web application (https://epimodel.shinyapps.io/covid-university/).

Emory University implemented a plan for Fall 2020 semester that included screening of all returning student, repeat screening of those residing on-campus. Staff, faculty and all students were also able to access optional screening using a rapid antigen test. In addition, testing of all symptomatic staff, students and faculty by PCR was prioritized. Up until time of writing (November 2020) the program was successful in that there was a moving 7-day average of about 1 staff/faculty and 1 student case per day and no large clusters were identified through October ^27^.

In conclusion, we present a model of SARS-CoV-2 transmission and control to assist universities in planning potential impacts and resource needs. Our model is conservative (meaning that it may overestimate the COVID burden on campus) in that we assume a high reproductive number that is not reduced through non-pharmaceutical interventions. Our study comes to a different conclusion than another model of COVID-19 control on university campuses ^5^. Paltiel et al. found that testing alone was unable to mitigate campus transmission and substantial disease control could only be achieved through the addition of frequent screening. However, that model did not include symptom-based surveillance leading to contact tracing efforts, as our model did. Contact tracing initiated by detection of cases can be highly impactful, but we also show that mitigation success required identifying a large proportion of contacts. Moreover, they did not model risk to faculty and staff, which we consider critical given their higher probability of severe disease and death owing to older age ^3^. In summary, we find that community-introduction of SARS-CoV-2 infection onto campus can be controlled with effective testing, isolation, contract tracing and quarantine, consistent with observations that this strategy has been successful in the general population where implemented properly (e.g., South Korea) ^28^.

## Supplementary Information

Supplementary Information.
