# Supplementary material for: A modeling study to inform screening and testing interventions for the control of SARS-CoV-2 on university campuses
Source: Sci Rep. 2021 Mar 15;11:5900. doi: 10.1038/s41598-021-85252-z (PMC7960702; doi:10.1038/s41598-021-85252-z)
Supplement: Supplementary file 1 — Supplementary Information. [file 41598_2021_85252_MOESM1_ESM.docx]

**A modeling study to inform screening and testing interventions for the control of SARS-CoV-2 on university campuses**

Ben Lopman, PhD^1*^;
Carol Y. Liu, MSc^1*^;
Adrien Le Guillou MD MPH^1,2^;
Andreas Handel PhD^3^;
Timothy L. Lash, DSc^1^;
Alexander P. Isakov MD MPH^4^;
Samuel M. Jenness, PhD^1^

**Affiliations**
^1^ Emory University Rollins School of Public Health, Atlanta, GA 30322, USA
^2^ Department of Research and Public Health, Reims Teaching Hospitals, Robert Debré Hospital, Reims, France
^3^ College of Public Health, University of Georgia, Athens, GA 30602, USA
^4^ Emory University School of Medicine, Atlanta, GA 30322, USA

*Contributed equally as co-1^st^ authors

**Correspondence**

Carol Liu

Emory University
1520 Clifton Road
Atlanta, GA 30323

Email: [carol.liu@emory.edu](mailto:carol.liu@emory.edu)

# Supplementary Information I. Model equations

$$\begin{matrix} \frac{dS_{i}}{dt} & =-\lambda_{i}S_{i}-com(S_{i})-\left[ \frac{1}{test}\left( 1-p_{{asy}_{i}} \right)I_{i}+ \frac{1}{scr}\left( I_{i}+E_{i} \right) \right]*{se}_{t}*(cont-Ro_{i})p_{cont}+\frac{1}{\delta}Q_{i}\left( cont-\frac{R0_{i}}{cont} \right) \\ \frac{dE_{i}}{dt} & =\lambda_{i}S_{i}+com*S_{i}-\frac{1}{\alpha}E_{i}-\frac{1}{scr}*{se}_{t}E_{i}-\left[ \frac{1}{test}\left( 1-p_{{asy}_{i}} \right)I_{i}+ \frac{1}{scr}\left( I_{i}+E_{i} \right) \right]*{se}_{t}*Ro_{i}*p_{cont} \\ \frac{dI_{i}}{dt} & =\frac{1}{\alpha}E_{i}-\frac{1}{\gamma}I_{i}-\frac{1}{scr}*{se}_{t}I_{t}-\frac{1}{test}*{se}_{t}(1-p_{{asy}_{i}})I_{i} \\ \frac{dP_{i}}{dt} & =\frac{1}{test}*{se}_{t}(1-p_{{asy}_{i}})I_{i}+\frac{1}{scr}*{se}_{t}(E_{i}+I_{i})-\delta P_{i} \\ \frac{dQ_{i}}{dt} & =\left[ \frac{1}{test}\left( 1-p_{{asy}_{i}} \right)I_{i}+ \frac{1}{scr}\left( I_{i}+E_{i} \right) \right]*{se}_{t}*(cont*p_{cont})-\frac{1}{\delta}Q_{i} \\ \frac{dR_{i}}{dt} & =\frac{1}{\gamma}I_{i}+\frac{1}{\delta}P_{i}+\frac{1}{\delta}Q_{i}\frac{Ro_{i}}{cont} \end{matrix}$$

Where i = on (students living on campus); off (students living off campus); saf (staff and faculty), and

$$\begin{matrix} \lambda_{on} & =(1-eff_{npi})(\beta_{stu,stu}(I_{on}+I_{off})+\beta_{on,on}I_{on}+\beta_{saf}I_{saf}) \\ \lambda_{off} & =(1-eff_{npi})(\beta_{stu,stu}(I_{on}+I_{off})+\beta_{saf}I_{saf}) \\ \lambda_{saf} & =(1-eff_{npi})(\beta_{saf}(I_{on}+I_{off}+I_{saf})) \end{matrix}$$

Table 1. Summary table of parameters, notations and mean value

| Parameter | Symbol | Mean Value |
| --- | --- | --- |
| Natural history and clinical |  |  |
| Latent period (days) | $\alpha$ | 3 |
| Infectious period (days) | $\gamma$ | 7 |
| Proportion severe - students | $p_{sev,stu}$ | 0.0224 |
| Proportion severe - staff/faculty | $p_{sev,saf}$ | 0.055 |
| Proportion fatal - students | $p_{death,stu}$ | 0.0006 |
| Proportion fatal - staff/faculty | $p_{death,saf}$ | 0.0052 |
| Proportion symptomatic - students | $1-p_{asy,stu}$ | 0.35 |
| Proportion symptomatic - staff/faculty | $1-p_{asy,saf}$ | 0.51 |
| Transmission |  |  |
| R_0_: students to students | ${Ro}_{stu,stu}$ | 2 |
| R_0_: on campus students to other on campus students | ${Ro}_{on,on}$ | 1 |
| R_0_: Staff/faculty to student; staff/faculty to staff/faculty | ${Ro}_{saf}$ | 0.5 |
| Daily per capita community infection | com | 0.0005 |
| Efficacy of face-coverings and social distancing | eff_npi | 0.35 |
| Testing and quarantine |  |  |
| Time from onset of infectiousness to testing (days) | test | 4 |
| Screening interval (days) | scr | 30 |
| Duration of quarantine or isolation (days) | δ | 14 |
| Number of contacts per case | cont | 2 |
| Proportion of contacts reached | $p_{cont}$ | 0.75 |
| Proportion experiencing ILI symptoms per day | ili | 0.00333 |
| PCR sensitivity on day t of infection (t=2,4,7) | ${se}_{t}$ | 0.75, 0.8, 0.75 |

# Supplementary Information IIa. Estimated active and cumulative cases under intervention scenarios with 25th and 75th centile range


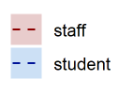


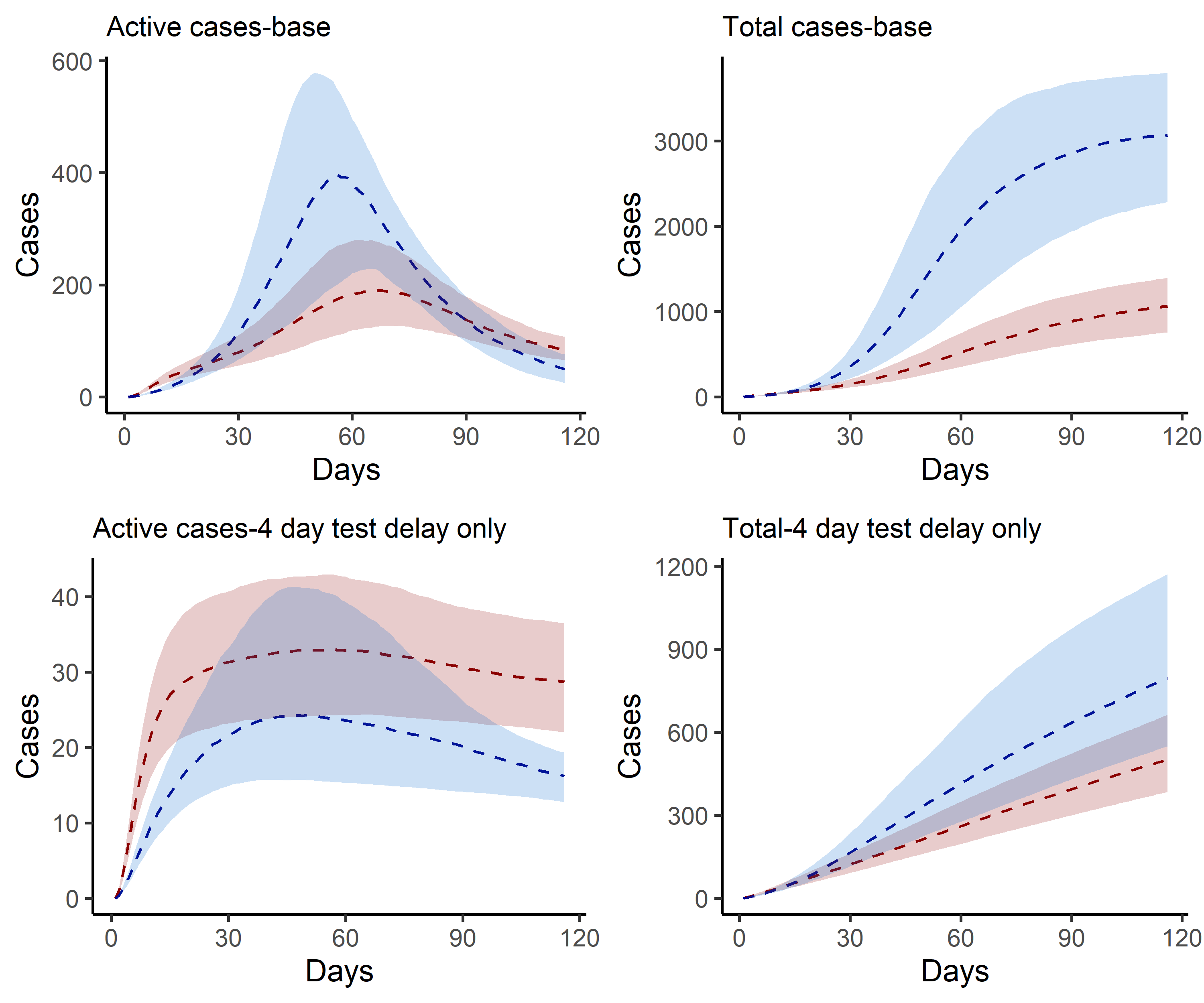


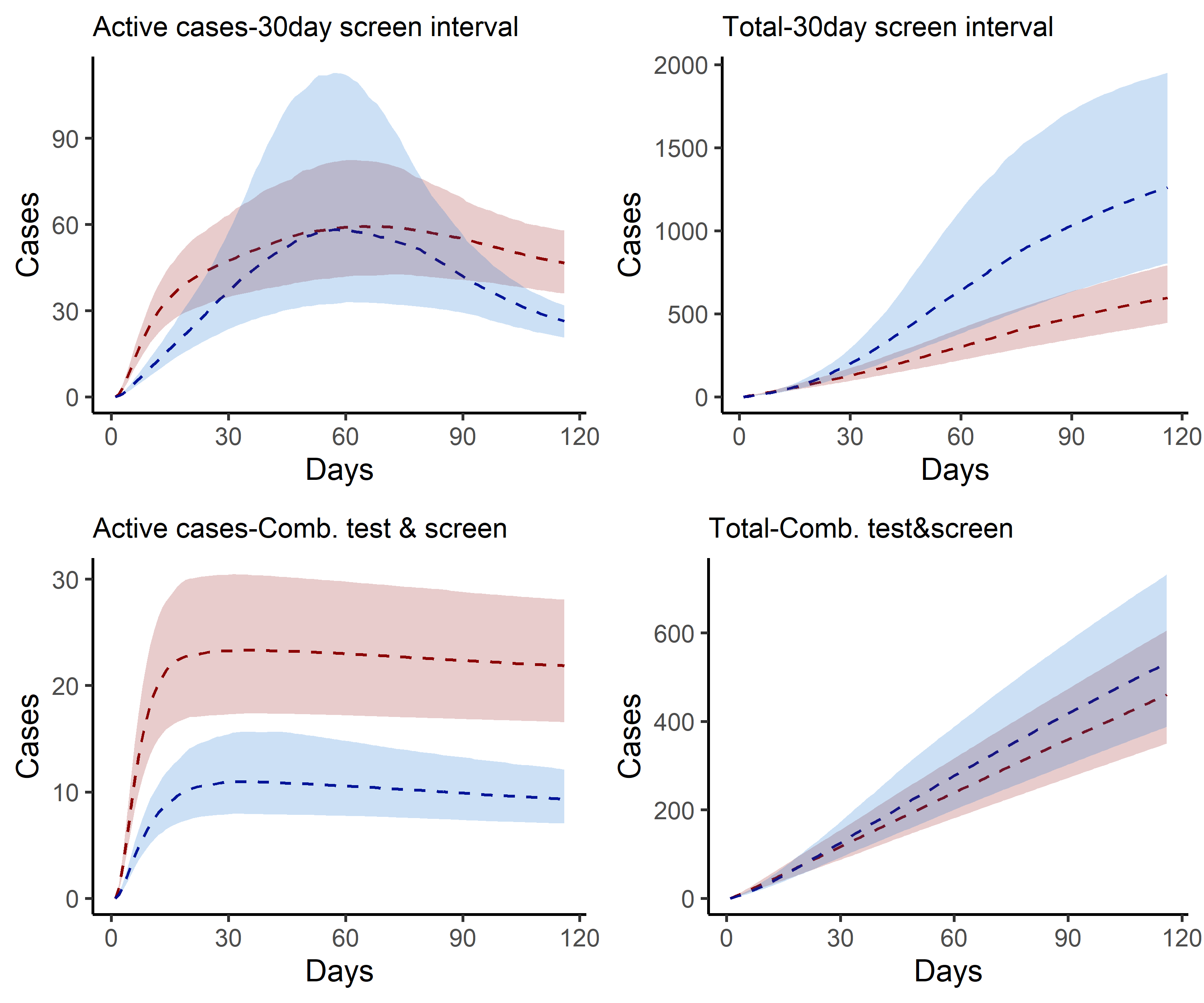


Figure 1: Active and cumulative student and staff cases under intervention scenarios based on probabilistic sensitivity analysis. The dotted line represents the median and shaded area represents the 25^th^-75^th^ centile range.

# Supplementary Information IIb. Table summarizing NPI, screening and testing interventions for model results presented in SI. IIa.

|  | **Base** | **4-day testing delay interval** | **30-day screening interval** | **Combined test & screen** |
| --- | --- | --- | --- | --- |
| NPI intervention efficacy | 35% | 35% | 35% | 35% |
| Screening interval | None | None | 30 days | 30 days |
| Testing interval | None | 4 days | None | 4 days |

# Table 2. Details on intervention scenarios for models presented in SI. IIa

# Supplementary Information III. Box plots visualizing the proportion of cumulative student and staff cases due to community transmission under different intervention scenarios


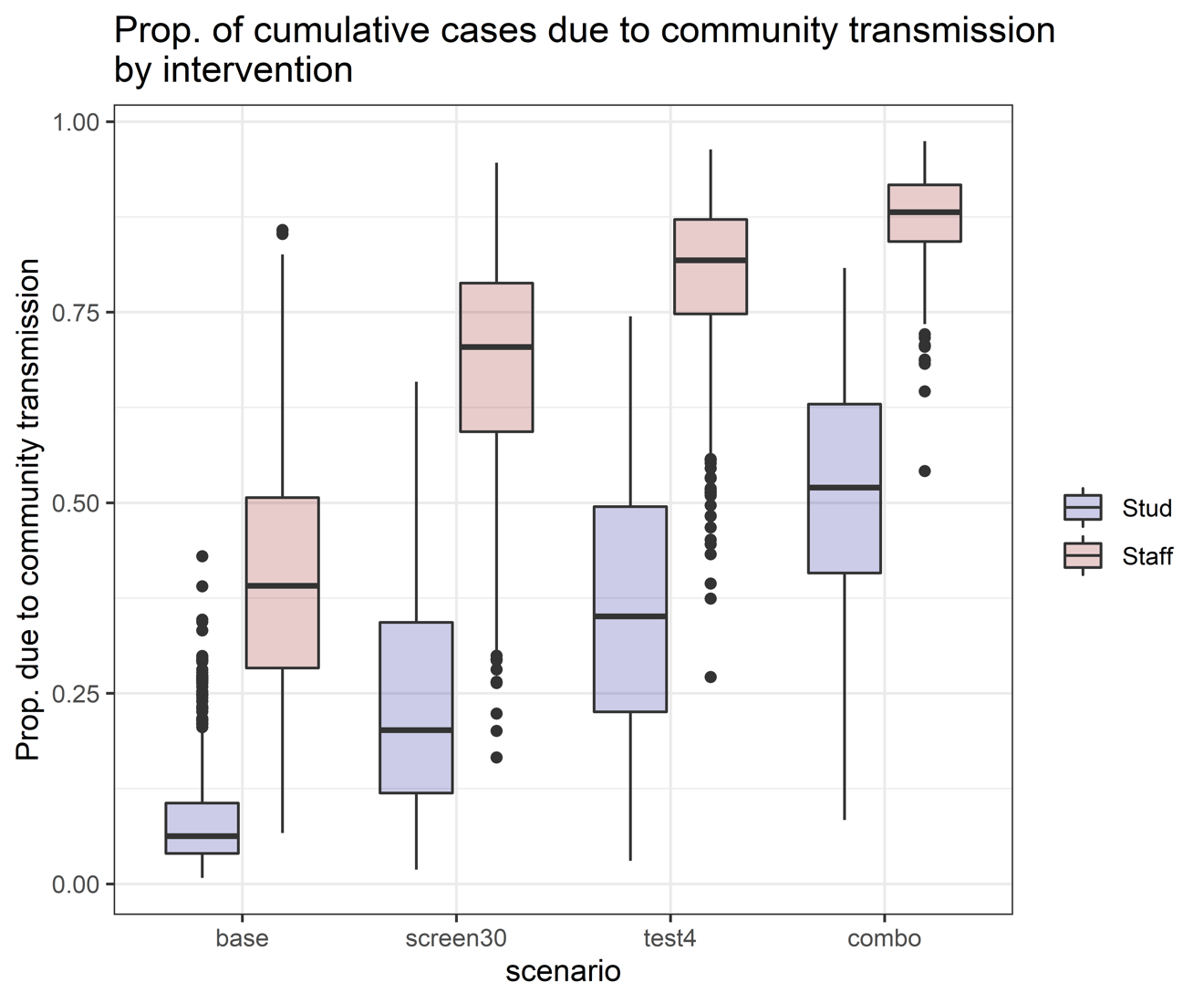


Figure 2. Box plots of proportion of cumulative student and staff cases due to community transmission under different intervention scenarios

**Supplementary Information IVa Pairwise scatterplots between model inputs and cumulative student cases using outputs from model with 30-day screening interval and 4-day testing delay**


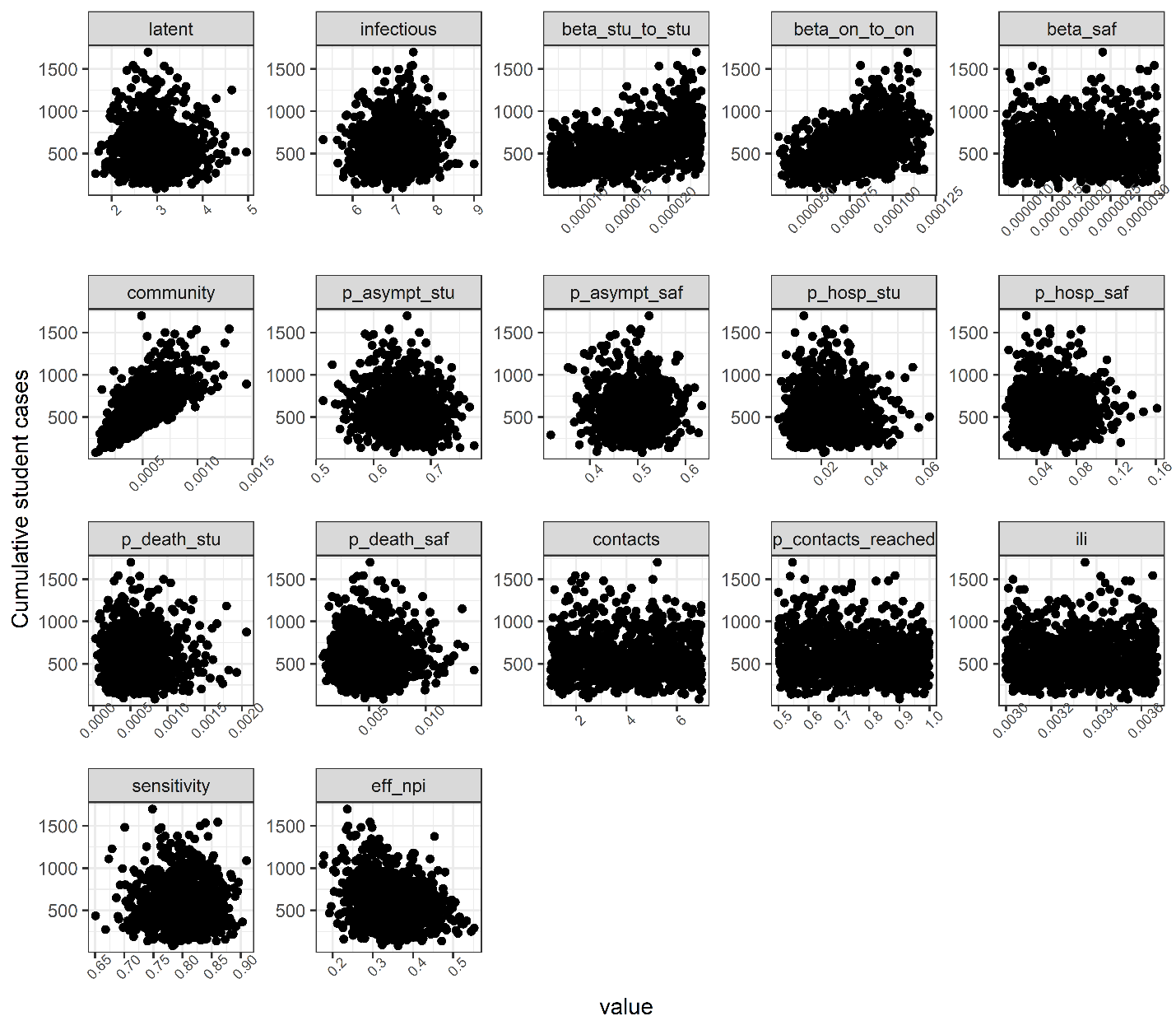


Figure 4. Pairwise scatterplots between model inputs and output of cumulative student cases

**Supplementary Information IVb Pairwise scatterplots between model inputs and cumulative staff cases using outputs from model with 30-day screening interval and 4-day testing delay**

**
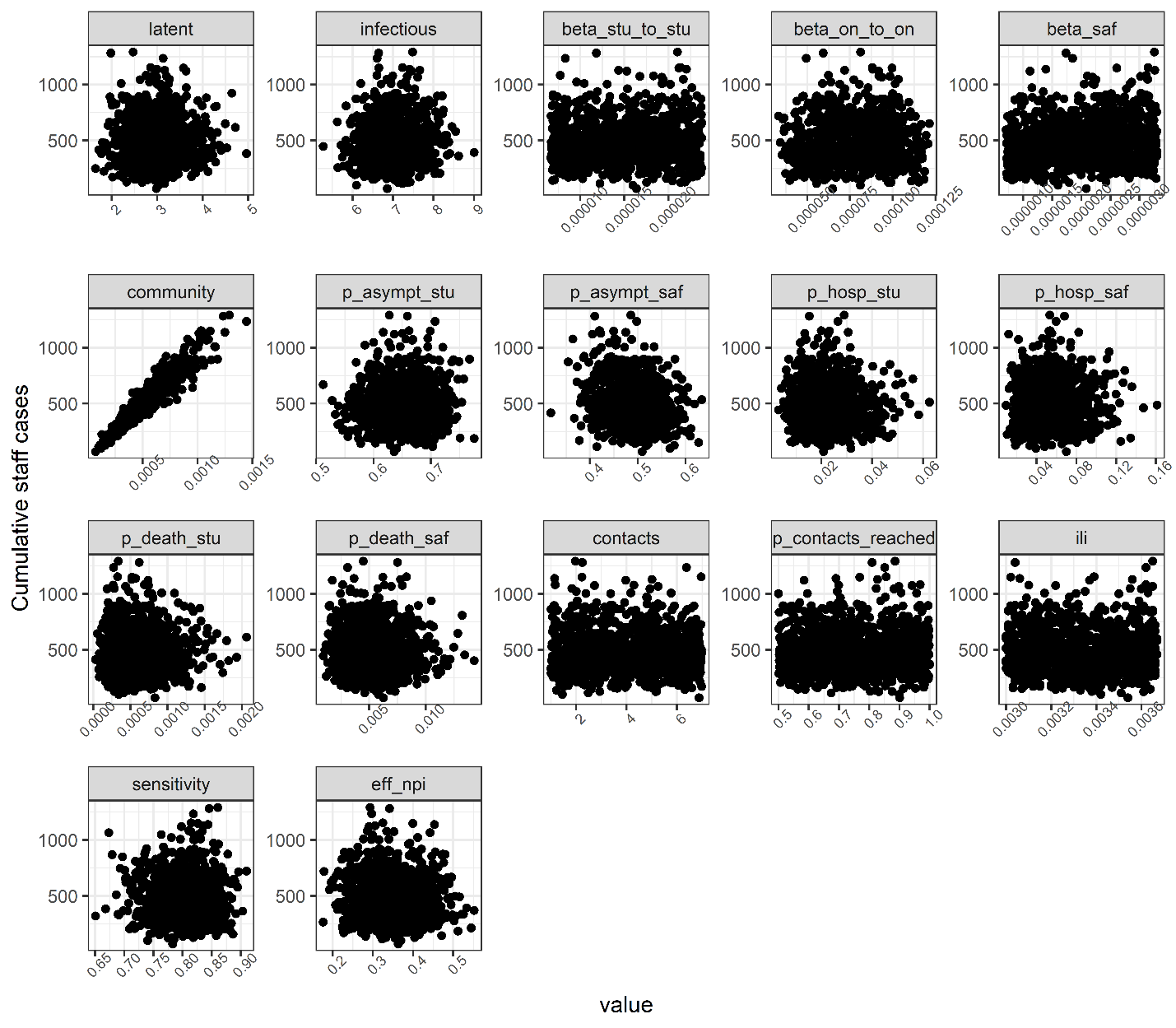
**

Figure 4. Pairwise scatterplots between model inputs and output of cumulative staff cases
